# ID 324 - Aperfeiçoamento Profissional para o Desenvolvimento de Diretrizes Clínico-Assistenciais para o Sistema de Saúde Público Brasileiro: descrição de um programa de ensino a distância

**DOI:** 10.5327/2237-9622.2025.v34s1.324

**Published:** 2025-11-25

**Authors:** Muriel Primon de Barros, Cintia Laura Pereira Araujo, Roseana Boek Carvalho, Marina Petrasi Guahnon, Ana Paula Blankenheim, Bárbara Cristiane da Silva, Bruna Marmett, Rodrigo Pereira de Almeida, Verônica Colpani, Ávila Vidal, Marta da Cunha Lobo Souto Maior, Cinara Stein, Gilson Pires Dorneles, Suena Medeiros Parahiba, Maicon Falavigna

**Keywords:** diretrizes clínico-assistenciais, programa de ensino à distância (EAD), Núcleos de Avaliação de Tecnologias em Saúde (Nats)

## Abstract

**Introdução:**

As diretrizes clínico-assistenciais são essenciais para a sustentabilidade do Sistema Único de Saúde (SUS) brasileiro, pois estabelecem padrões e recomendações baseados em evidências para o diagnóstico e o tratamento de condições de saúde. Tutorias e programas de capacitação possuem potencial para conferir suporte metodológico a profissionais envolvidos nessas atividades, consequentemente melhorando a padronização e o desenvolvimento técnico-científico de diretrizes. O objetivo deste estudo foi descrever um programa de ensino a distância (EAD) para o aprimoramento profissional no desenvolvimento de diretrizes para o SUS.

**Método:**

O programa em EAD promoveu cinco cursos entre 2020 e 2023: 1) curso básico síncrono e supervisionado (CBSS), com foco no desenvolvimento de diretrizes clínico-assistenciais (duas edições); 2) curso básico auto-instrucional (Cbai); 3) curso avançado síncrono (CAS) sobre diretrizes clínico-assistenciais; 4) curso síncrono sobre o sistema GRADE (CSG) (duas edições); e 5) curso autoinstrucional sobre o sistema GRADE (CAIG), voltados para profissionais dos Núcleos de Avaliação de Tecnologias em Saúde (Nats) envolvido no desenvolvimento de diretrizes junto ao Ministério da Saúde.

**Resultados:**

Profissionais de 13 Nats participaram do programa EAD. O CBSS e o Cbai foram oferecidos em 16 turmas (8 e 7 módulos, respectivamente). Quarenta e oito participantes concluíram o CBSS, com uma média de presença de 80% (nota final média: 8,6±2,0). No Cbai, 263 participantes de 20 estados brasileiros concluíram o curso (média final: 8,3±1,0). O CAS ofereceu 9 aulas síncronas semanais (8 módulos) e foi concluído por 37 participantes de 11 Nats (nota final: 9,3±1,2). Duas edições do CSG foram oferecidas em 2021 e 2022, durante 8 semanas, com 14 e 16 aulas assíncronas e síncronas, respectivamente (6 módulos). Quarenta e oito participantes de 11 Nats completaram o curso, com uma média de presença de 86% (nota final: 7,7±2,7). No CAIG, 13 turmas (7 módulos) foram disponibilizadas, com 115 participantes de 15 estados brasileiros(média final de 8,7±1,1). Para ações futuras, os participantes sugeriram, em suas avaliações sobre o programa, a inclusão de tópicos relacionados ao desenvolvimento de diretrizes clínico-assistenciais e avaliações econômicas em saúde visando à evolução contínua.

**Conclusão:**

O programa EAD mostra-se uma potencial estratégia para aprimorar a capacidade profissional no desenvolvimento de diretrizes clínico-assistenciais por meio de práticas baseadas em evidências no apoio à tomada de decisão no Brasil. A flexibilidade da modalidade on-line permitiu a participação de profissionais de diferentes regiões, superando barreiras geográficas e promovendo uma troca de conhecimentos mais ampla e diversificada.

